# Three-dimensional analysis of synapses in the transentorhinal cortex of Alzheimer’s disease patients

**DOI:** 10.1186/s40478-018-0520-6

**Published:** 2018-03-02

**Authors:** M. Domínguez-Álvaro, M. Montero-Crespo, L. Blazquez-Llorca, R. Insausti, J. DeFelipe, L. Alonso-Nanclares

**Affiliations:** 1 Laboratorio Cajal de Circuitos Corticales, Centro de Tecnología Biomédica, Universidad Politécnica de Madrid, Pozuelo de Alarcón, 28223 Madrid, Spain; 2 Instituto Cajal, Consejo Superior de Investigaciones Científicas (CSIC), Avda Doctor Arce, 37, 28002 Madrid, Spain; 3 Centro de Investigación Biomédica en Red sobre Enfermedades Neurodegenerativas (CIBERNED), ISCIII, Madrid, Spain; 4 Laboratorio de Neuroanatomía Humana, Facultad de Medicina, Universidad de Castilla-La Mancha, Almansa 14, 02006 Albacete, Spain; 5 Depto. Psicobiología, Facultad de Psicología, Universidad Nacional de Educación a Distancia (UNED), c/Juan del Rosal, 10, 28040 Madrid, Spain

**Keywords:** Dementia, Electron Microscopy, FIB/SEM, Medial temporal lobe, Neuropil, Synapses

## Abstract

Synaptic dysfunction or loss in early stages of Alzheimer’s disease (AD) is thought to be a major structural correlate of cognitive dysfunction. Early loss of episodic memory, which occurs at the early stage of AD, is closely associated with the progressive degeneration of medial temporal lobe (MTL) structures of which the transentorhinal cortex (TEC) is the first affected area. However, no ultrastructural studies have been performed in this region in human brain samples from AD patients.

In the present study, we have performed a detailed three-dimensional (3D) ultrastructural analysis using focused ion beam/scanning electron microscopy (FIB/SEM) to investigate possible synaptic alterations in the TEC of patients with AD. Surprisingly, the analysis of the density, morphological features and spatial distribution of synapses in the neuropil showed no significant differences between AD and control samples. However, light microscopy studies showed that cortical thickness of the TEC was severely reduced in AD samples, but there were no changes in the volume occupied by neuronal and glial cell bodies, blood vessels, and neuropil. Thus, the present results indicate that there is a dramatic loss of absolute number of synapses, while the morphology of synaptic junctions and synaptic spatial distribution are maintained. How these changes affect cognitive impairment in AD remains to be elucidated.

**Electronic supplementary material:**

The online version of this article (10.1186/s40478-018-0520-6) contains supplementary material, which is available to authorized users.

## Introduction

Alzheimer’s Disease (AD) is the main cause of dementia, accounting for 60−80% of cases in the adult population [3]. The disease is characterized by a progressive and persistent decline of cognitive functions, such as memory and orientation. In the final stages, patients suffer a severe lack of autonomy and social life [56]. AD is characterized by two hallmark lesions: extracellular amyloid plaques, primarily consisting of amyloid-β (Aβ) peptide, and intracellular neurofibrillary tangles, which consist of filamentous aggregates of hyperphosphorylated tau protein. In addition to these main hallmarks of AD, other neuropathological changes —such as neuronal and synaptic loss— have also been described [58]. Among them, synaptic loss seems to be the major structural correlate of cognitive decline observed in AD patients, and it is considered to be the earliest mechanism that precedes neuronal loss [5, 18, 23, 43, 70, 82].

Even though it seems that AD begins in subcortical regions [12, 13, 15], the early loss of episodic memory is closely associated with the progressive degeneration of the medial temporal lobe (MTL) structures [71]. Among the MTL structures, the transentorhinal cortex (TEC) is one of the first affected areas [11]. This obliquely oriented cortex is located in the MTL between the perirhinal cortex (PRC) and the entorhinal cortex (EC) [10, 14]. The TEC is considered as a transitional zone between the periallocortex represented by EC and the proisocortex [10, 14, 36]. Although the TEC is not considered as a distinct area in Brodmann’s nomenclature, it is nonetheless part of area 35 (perirhinal cortex), which —together with Brodmann’s area 36 (ectorhinal cortex)— is nowadays frequently named perirhinal cortex (PRC) [42].

Obvious ethical reasons prevent tract-tracing connectivity studies on human brain other than in post-mortem studies; however, studies performed in monkey have shown that the PRC constitutes a convergence zone, receiving two main inputs. First, it connects extensively with MTL structures, including (in order of decreasing strength): EC, parahippocampal cortex, amygdala and hippocampus. Secondly, it has connections with cortical association areas (visual, insula, temporal, cingulate and orbitofrontal) [79, 81]. One of the main MTL reciprocal connections is established with the EC: inputs terminate in layer II and layers V−VI of the PRC (area 35 and the medial portion of area 36) [80, 81], while the main output of PRC is directed to layer I and III of the EC [35]. These strong interconnections make PRC unique, occupying a key position where sensory information converges with other information from memory-related structures, integrating memory information [78, 81]. Furthermore, it has been reported that neurons in layer II of the TEC are affected by neurofibrillary degeneration in primary age-related tauopathy [11, 72]. Thus, investigating the changes that affect synaptic connectivity in the TEC, as an early affected area in AD, is essential to better understand the mechanisms underlying this disease. As far as we know, no detailed ultrastructural studies have been performed in the human TEC.

Therefore, in order to investigate possible microanatomical changes related to this pathological condition, we performed an ultrastructural study of layer II neuropil of the TEC in human brain samples from AD patients and subjects with no apparent neurological alterations. We used focused ion beam/scanning electron microscopy (FIB/SEM) and three-dimensional (3D) reconstructions [45] to determine the density, types and features of the synapses, as well as their spatial distribution. Moreover, since it has been reported that there is a reduction in the thickness of frontal cortex, temporal cortex (areas 21 and 22 of Brodmann) and in the molecular layer of dentate gyrus (DG) in AD patients [59, 60, 63, 69], light microscopy studies were performed to evaluate the decrease in TEC thickness. Additionally, the same TEC samples were examined with light microscopy to investigate the volume occupied by neurons, glia, blood vessels and neuropil in both control and AD patients.

## Materials and methods

### Tissue preparation

Human brain tissue was obtained from three sources: Pathological Anatomy Service of Bellvitge University Hospital (Barcelona, Spain), *Centro Alzheimer Fundación Reina Sofía,* CIEN Foundation (Madrid, Spain) and Human Neuroanatomy Laboratory, School of Medicine, University of Castilla-La Mancha (Albacete, Spain).

Samples were obtained from five subjects with no apparent neurological alterations and from five patients with AD. According to the neuropathological criteria provided by the above-mentioned centers, cases were classified as control (subjects with no apparent neurological alterations) or AD (Table 1). Brain tissue samples were obtained following the guidelines and approval of the Institutional Ethical Committee. In all cases, the time between death and tissue processing was lower than 4 h.

**Table 1:** Clinical and neuropathological information

Patient	Gender	Age (years)	Cause of death	Postmortem delay (h)	Braak Stage	CERAD Stage	Neuropsychological diagnosis
AB1	Male	45	Lung cancer	< 1	NA	NA	NA
AB2	Female	53	Pulmonary shock	4	NA	NA	NA
IF10	Male	66	Bronchopneumonia plus cardiac failure	2	NA	NA	NA
M16	Male	40	Traffic accident	3	NA	NA	NA
M17	Male	36	Bronchopneumonia	2.5	NA	NA	NA
IF1	Female	80	–	2	IV	B	No evidence of cognitive impairment and dementia
IF2	Female	94	Pulmonary tuberculosis	1.5	V	C	Dementia
IF6	Male	85	Pneumonia	2	III	A	Mild cognitive impairment
VK11	Female	87	Respiratory inflammation	1.5	III−IV	A	Dementia
VK22	Female	86	–	2	V	C	Dementia

*NA* Not applicable, *NFTs* neurofibrillary tangles, - Not available

Braak Stages [11]: III (NFTs in entorhinal cortex and closely related areas); III−IV (NFTs abundant in amygdala and hippocampus. Extending slightly into association cortex); V−VI (NFTs widely distributed throughout the neocortex and ultimately involving primary motor and sensory areas). CERAD Stages [48]: A (Low density of neuritic plaques); B (Intermediate density of neuritic plaques); C (High density of neuritic plaques)

Upon removal, brain tissue was fixed in cold 4% paraformaldehyde (Sigma-Aldrich, St Louis, MO, USA) in 0.1 M sodium phosphate buffer (PB; Panreac, No.131965, Spain) pH 7.4 for 24−48h. After fixation, the tissue was washed in PB and sectioned coronally in a vibratome (Vibratome Sectioning System, VT1200S Vibratome, Leica Biosystems, Germany).

### Immunohistochemistry

Selected sections were first rinsed in PB 0.1 M, pretreated in 2% H_2_O_2_ for 30 min to remove endogenous peroxidase activity, and then incubated for 1 h at room temperature in a solution of 3% normal horse serum (for polyclonal antisera and monoclonal antibodies, respectively; Vector Laboratories Inc., Burlingame, CA) and 0.25% Triton-X (Merck, Darmstadt, Germany). Subsequently, sections were incubated for 48 h at 4 °C in the same solution with mouse anti-NeuN (1:2000; Chemicon; MAB377, Temecula, CA, USA) and anti-human PHF_-Tau_ antibody clone AT8 (1:2000, MN1020, Thermo Scientific, Waltham, MA, USA); for the sake of clarity, we will refer to this as anti-PHF_-Tau-AT8_. Sections selected for anti-Aβ were first treated with 88% formic acid (Sigma-Aldrich, No. 251364, St. Louis, MO, USA) to ensure specific plaque immunostaining, and were then incubated in a solution containing mouse antibody anti-Aβ (clone 6F/3D; 1:50, Dako M0872, Glostrup, Denmark). Sections were then processed with a secondary biotinylated horse anti-mouse IgG antibody (1:200, Vector Laboratories, Burlingame, CA, USA), and then incubated for 1 h in an avidin-biotin peroxidase complex (Vectastain ABC Elite PK6100, Vector) and, finally, with the chromogen 3,3′-diaminobenzidine tetrahydrochloride (DAB; Sigma-Aldrich, St. Louis, MO, USA). Finally, sections were dehydrated, cleared with xylene and cover-slipped.

### Electron microscopy

**Fig. 1:**
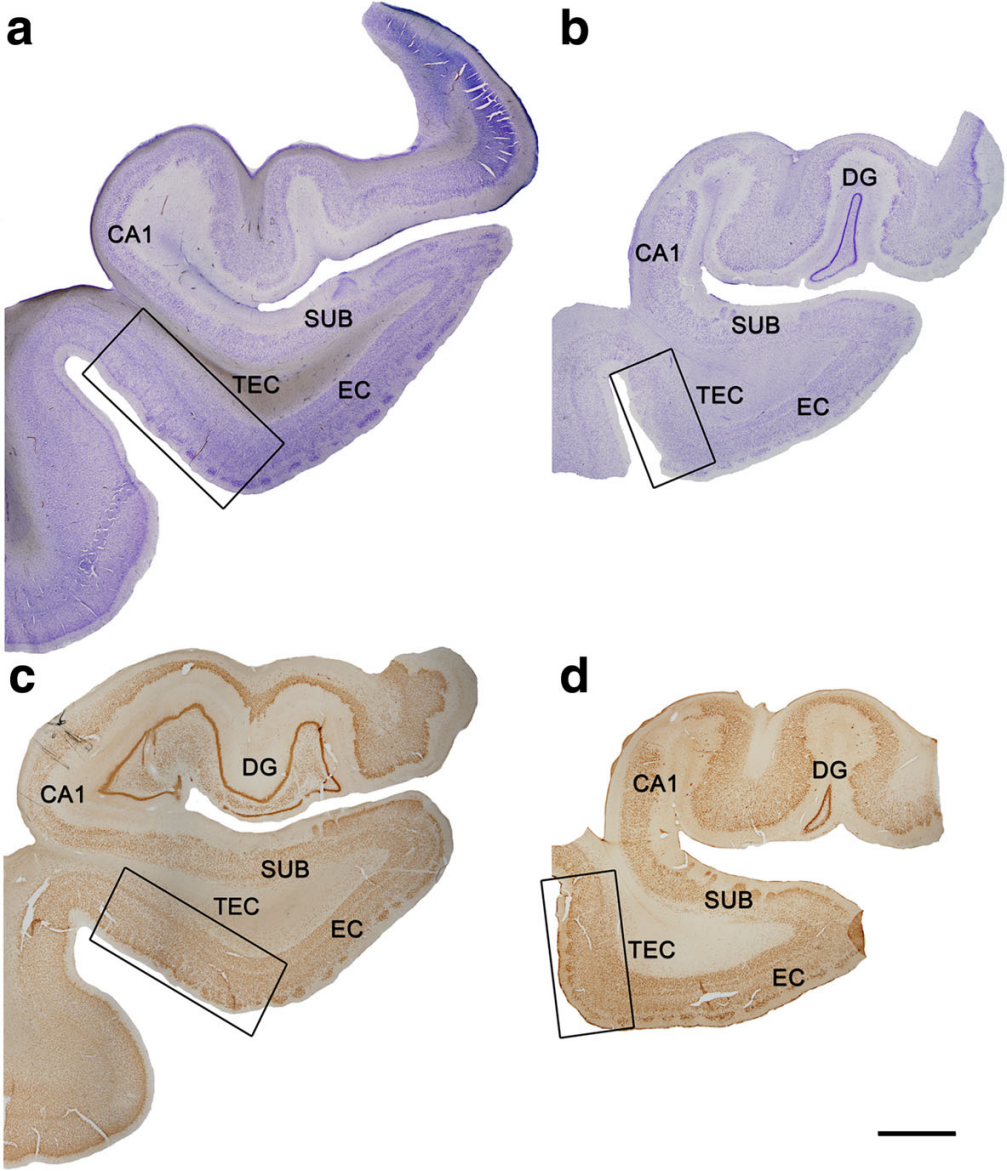
Coronal sections of human hippocampal formation. Low-power photographs of a control subject (**a**, **c**) and an AD patient (**b**, **d**), in sections stained for Nissl (**a**, **b**) and immunostained for anti-NeuN (**c**, **d**). TEC is indicated by the box. Scale bar (in **d**): 3 mm

**Fig. 2:**
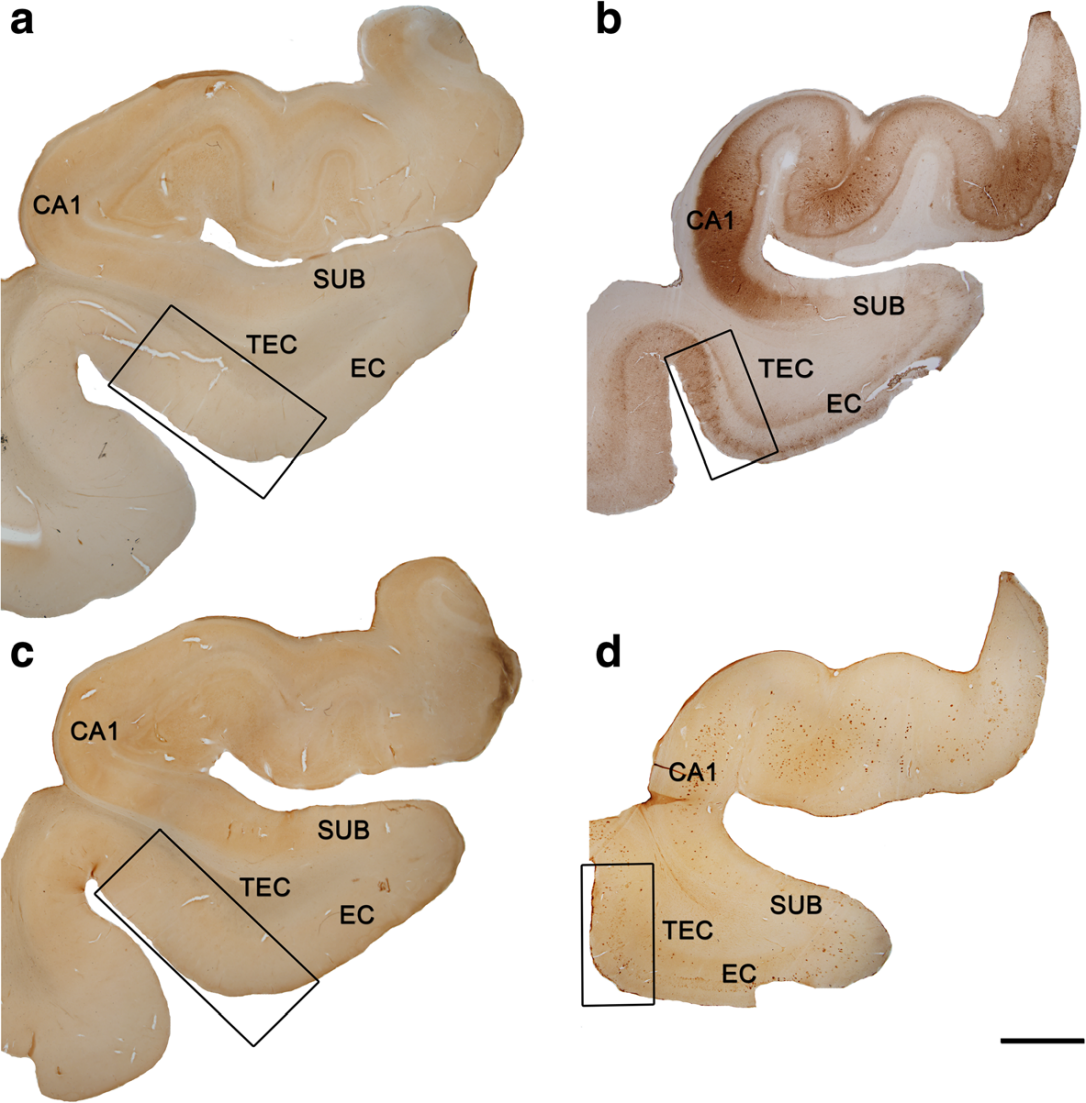
Coronal sections of human hippocampal formation. Low-power photographs of a control subject (**a**, **c**) and an AD patient (**b**, **d**), in sections immunostained for anti-PHF_-Tau-AT8_ (**a**, **b**) and anti-Aβ (**c**, **d**). TEC is indicated by the box. Immunostaining for anti-PHF_-Tau-AT8_ (**b**) and anti-Aβ (**d**) can be observed in the AD patient. These neuropathological marks are absent in the control subject (**a**, **c**). Scale bar (in **d**): 3 mm

**Fig. 3:**
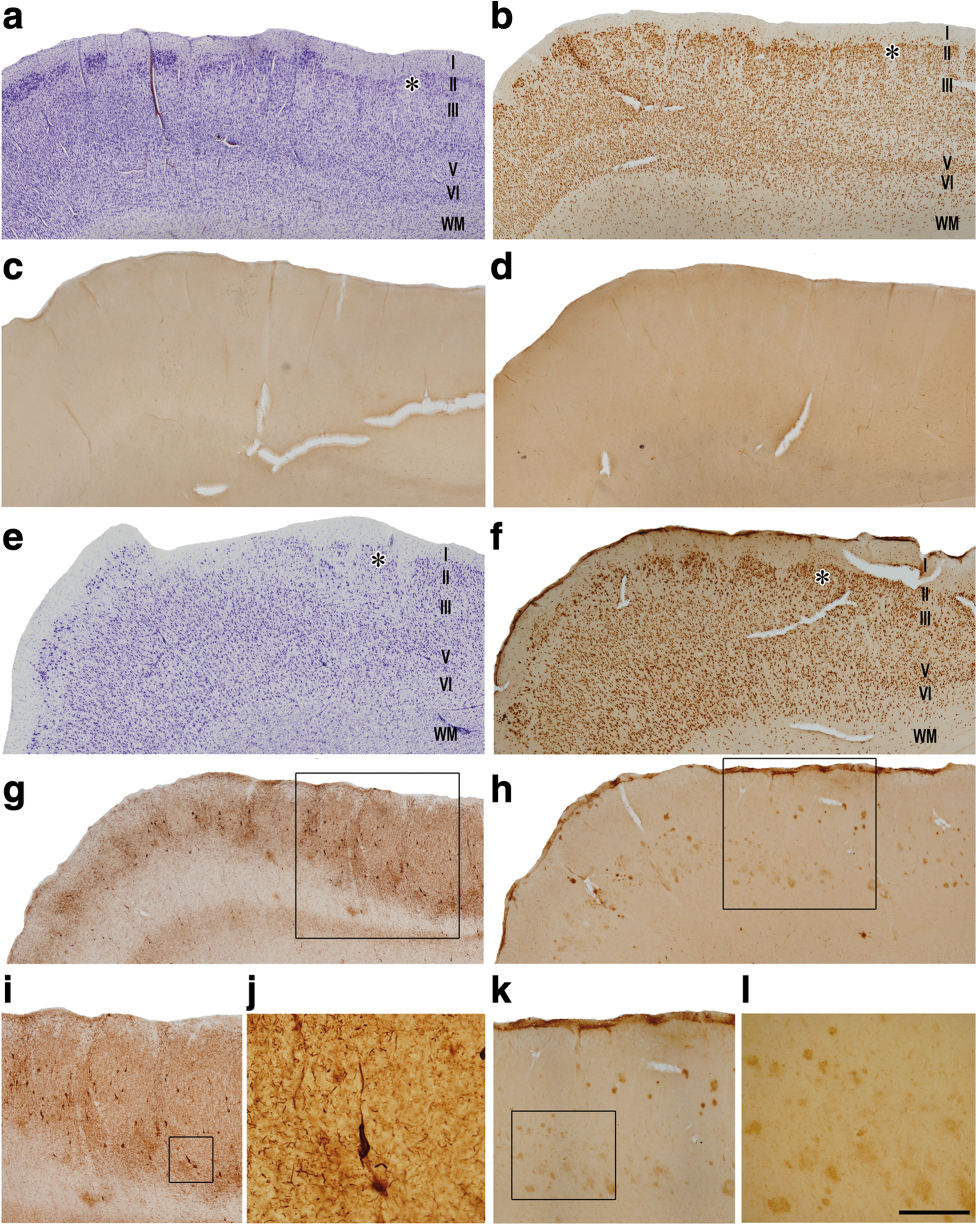
Higher magnifications of human TEC in coronal sections. Series of photomicrographs from a control subject (**a**−**d**) and an AD patient (**e**−**l**). Sections are stained for Nissl (**a**, **e**), and immunostained with antibodies anti-NeuN (**b**, **f**), anti-PHF_-Tau-AT8_ (**c**, **g**, **i**, **j**) and anti-Aβ (**d**, **h**, **k**, **l**). PHF_-Tau-AT8_ positive neurons (**g**, **i**, **j**) and Aβ positive plaques (**h**, **k**, **l**) are observed in TEC from the AD patient. Scale bar (in panel **l**) corresponds to: 1 mm in panels **a-d**; 800 μm in panels **e-h**; 530 μm in panels **i** and **k**; 110 μm in panel **j**, and 220 μm in panel **l**

Sections containing TEC were selected (Figs. 1, 2, and 3; Table 1) and postfixed for 24 h in a solution containing 2% paraformaldehyde, 2.5% glutaraldehyde (TAAB, G002, UK) and 0.003% CaCl_2_ (Sigma, C-2661-500G, Germany) in sodium cacodylate (Sigma, C0250-500G, Germany) buffer (0.1 M). These sections were washed in sodium cacodylate buffer (0.1 M) and treated with 1% OsO_4_ (Sigma, O5500, Germany), 0.1% potassium ferrocyanide (Probus, No. 23345, Spain) and 0.003% CaCl_2_ in sodium cacodylate buffer (0.1 M) for 1 h at room temperature. After washing in PB, sections were stained with 2% uranyl acetate (EMS, 8473, USA), and then dehydrated and flat-embedded in Araldite (TAAB, E021, UK) for 48 h at 60 °C [20]. Embedded sections were glued onto a blank Araldite block and trimmed. Semithin sections (1–2 μm thick) were obtained from the surface of the block and stained with 1% toluidine blue (Merck, No.115930, Germany) in 1% sodium borate (Panreac, No. 141644, Spain). The last semithin section (which corresponds to the section immediately adjacent to the block surface) was examined under light microscope and photographed to accurately locate the neuropil regions to be examined.

### Tissue shrinkage estimation

Tissue shrinkage due to electron microscopy processing was estimated measuring the area before and after processing to correct the final values in both control and AD cases [45]. The area after processing was divided by the area value measured before processing, to obtain a shrinkage factor for any area measurement (p^2^) of 0.933.

Moreover, to estimate differences between control and AD cases, we measured the cortical thickness of TEC in three to five toluidine blue-stained semithin sections from all cases, obtained in the coronal plane of the cortex and containing the entire cortex, from the pial surface to the white matter. Measurements of the distance between the pial surface and the boundary with the white matter were performed with the aid of Fiji program (ImageJ 1.51; NIH, USA; http://imagej.nih.gov/ij/). To average data, three measurements were made per section.

In addition, FIB/SEM stacks of images were also corrected for the presence of fixation artifacts, which did not affect the proper identification and quantitation of synapses, i.e., swollen neuronal or glial processes. The volume occupied by these artifacts, calculated applying the Cavalieri principle [30], was discounted from the volume of the stacks of images to avoid under-estimation of the number of synapses per volume. Every FIB/SEM stack was examined and the volume artifact ranged between 3 and 33% of the volume stacks. Data on the number of synapses per volume were corrected accordingly.

### Volume fraction estimation of cortical elements

Three to five semithin sections (1–2 μm thick) from all cases stained with toluidine blue were used to estimate the respective volume fractions occupied by (i) neuropil, (ii) cell bodies (from neurons and glia) and (iii) blood vessels. This estimation was performed applying the Cavalieri principle [30] by point counting using the integrated Stereo Investigator stereological package (Version 8.0, MicroBrightField Inc., VT, USA) attached to an Olympus light microscope (Olympus, Bellerup, Denmark) at 40× magnification. A grid, whose points covered an area of 400μm^2^, was overlaid over each semithin section to determine the volume fraction (V_v_) occupied by the different elements: neurons, glia, blood vessels and neuropil (Additional file 1: Figure S1A). V_v_ was estimated with the following formulae: V_v_ neuropil = 100 - (V_v_ neurons+ V_v_ glia + V_v_ blood vessels).

### Three-dimensional electron microscopy

The 3D study of the samples was carried out using a dual beam microscope (Crossbeam® Neon40 EsB, Carl Zeiss NTS GmbH, Oberkochen, Germany). This instrument combines a high-resolution field-emission SEM column with a focused gallium ion beam (FIB), which permits removal of thin layers of material from the sample surface on a nanometer scale. As soon as one layer of material is removed by the FIB (20 nm thick), the exposed surface of the sample is imaged by the SEM using the backscattered electron detector. The sequential automated use of FIB milling and SEM imaging allowed us to obtain long series of photographs of a 3D sample of selected regions [45]. Image resolution in the xy plane was 5 nm/pixel. Resolution in the z axis (section thickness) was 20 nm, and image size was 2048 × 1536 pixels. Although the resolution of FIB/SEM images can be increased, we have chosen these parameters as a compromise solution to obtain a large enough field of view where synaptic junctions could still be clearly identified, in a period of time that allowed us to have long series of sections in a relatively short, reasonable time (approximately 12 h per stack of images). The number of sections per stack ranged from 149 to 472, which corresponds to a corrected volume ranging from 260.2 to 824.4μm^3^ (mean: 471.3μm^3^). A total of 30 stacks of images of the neuropil from layer II of the TEC were obtained (three stacks per case, in all 10 cases; total volume studied: 14,140μm^3^).

### Synaptic three-dimensional analysis

**Fig. 4:**
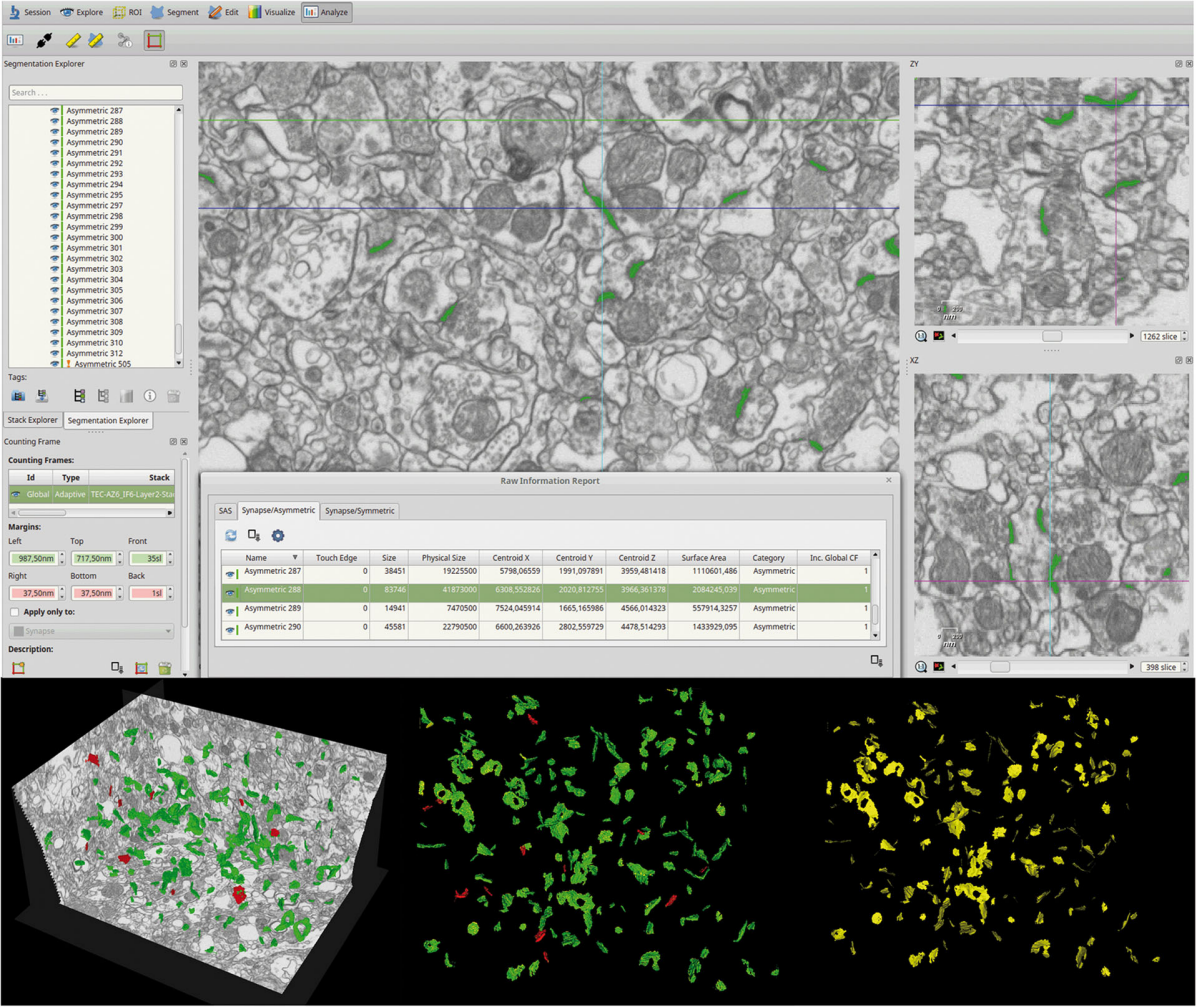
Screenshot of the EspINA software user interface. In the main window (top), the sections are viewed through the xy plane (as obtained by the FIB/SEM microscopy). The other two orthogonal planes, yz and xz, are also shown in adjacent windows. 3D reconstruction of a synapse is shown in the three orthogonal planes. The “Raw Information Report” window shows features extracted from the reconstructed 3D synapse. The 3D windows (bottom) show the three orthogonal planes and the 3D reconstruction of segmented synapses (bottom left). Synapses appear green (asymmetric synapses) or red (symmetric synapses) according to the colors assigned by the user, and their SAS appear yellow (bottom right)

Stacks of images obtained by the FIB/SEM were analyzed using EspINA software (*EspINA Interactive Neuron Analyzer*, 2.1.9; http://cajalbbp.cesvima.upm.es/espina/), which allows the segmentation of synapses in the reconstructed 3D volume (for a detailed description of the segmentation algorithm, see [49]; Fig. 4). Since the synaptic junctions were fully reconstructed as described elsewhere [45], each synapse could be classified as asymmetric (AS) or symmetric (SS) based on its prominent or thin post-synaptic density (PSD: Additional file 1: Figure S2), respectively [29, 53].

EspINA provided the number of synapses in a given volume, which allows the estimation of the number of synapses per volume. EspINA also allowed the application of an unbiased 3D counting frame to perform direct counting (for details, see [45]).

In addition, geometrical features —such as size and morphology— and spatial distribution features (centroids) of each reconstructed synapse were also calculated by EspINA. This software also extracts the Synaptic Apposition Surface (SAS) and provides its morphological properties. Since the pre- and post-synaptic densities are located face to face, their surface areas are comparable (for details, see [50]). Since the SAS adapts to the curvature of the synaptic junction we have also measured its curvature as one minus the ratio between the projected area of the SAS and the area of the SAS. This measurement would be 0 in a flat SAS, and would increase to a maximum of 1 as the SAS curvature increases. Since the SAS comprises both the active zone and the PSD, it is a functionally relevant measure of the size of a synapse (Fig. 4) [50].

### Spatial distribution analysis of synapses

To analyze the spatial distribution of synapses, Spatial Point Pattern analysis was performed as described elsewhere [4, 46]. Briefly, we compared the actual position of centroids of synapses with the Complete Spatial Randomness (CSR) model — a random spatial distribution model which defines a situation where a point is equally likely to occur at any location within a given volume. For each of the 30 different samples, we calculated three functions commonly used for spatial point pattern analysis: G, F and K functions (for a detailed description, see [9]). An additional step to explore the spatial distribution of a spatial pattern is to obtain the distance to the nearest neighbor. To do this, the distance of each synapse to its nearest synapse was measured, and comparison between control and AD patients was also performed. This study was carried out using the Spatstat package and R Project program [6].

### Statistical analysis

To determine possible differences between groups, statistical comparisons of synaptic density, proportion of synapses, TEC thickness, synaptic size (SAS), neuronal and glial cell bodies, blood vessels and neuropil volume fraction, as well as the distance to the nearest neighbor were carried out using the unpaired Mann-Whitney (MW) nonparametric U-test (the normality and homoscedasticity criteria were not met). Frequency distribution analysis of the SAS was performed using Kolmogorov-Smirnov (KS) nonparametric test. Statistical studies were performed with the aid of the GraphPad Prism statistical package (Prism 5.00 for Windows, GraphPad Software Inc., USA) and SPSS program (IBM SPSS Statistics v22, IBM Corp., USA).

## Results

### Histopathological findings

The TEC region was delimited on the basis of previous studies [10, 14]. In Nissl-stained sections and sections immunostained for anti-NeuN, the TEC was distinguished because layers III and V merge and sweep obliquely to invade layer II of the EC [10, 14, 24, 85]. TEC is considered as part of the PRC area 35. PRC is proisocortex [36] and lacks a layer IV (agranular type of cortex). The most typical feature of TEC is the presence of an oblique band of neurons between layers V and III [24, 37] of PRC. Standard histopathological assessment of all cases was performed on Nissl-stained and NeuN-immunostained sections containing the TEC. AD cases showed an apparent reduction in the total volume of MTL structures, including TEC (Figs. 1, 2). In addition, immunostaining for anti-PHF-_Tau_ and anti-Aβ revealed the presence of a variable amount of immunoreactive PHF-_Tau_ neurons and Aβ-plaques in the AD cases, whereas in control cases no Aβ-plaques were found and only occasional PHF-_Tau_ neurons were present (Figs. 1, 2, and 3; Table 1).

To evaluate the degree of reduction, measurements of the whole thickness of TEC were performed in toluidine blue-stained semithin sections. These results revealed a significantly lower thickness of the TEC (35% lower) in AD patients compared to controls (MW, *p* = 0.02; Table 2; Additional file 1: Table S1); its average thickness was 1.74 mm in AD patients versus 2.66 mm in control subjects.

**Table 2:** Volume fraction occupied by cortical elements in layer II of the TEC. All volume data are corrected for shrinkage

Group	V_neu_ (%; mean ± SD)	V_g_ (%; mean ± SD)	V_bv_ (%; mean ± SD)	V_n_ (%; mean ± SD)	TEC thickness (mm; mean ± SD)
Control	7.17 ± 0.98	0.50 ± 0.14	3.28 ± 0.41	89.05 ± 1.22	2.66 ± 0.37
Alzheimer	5.86 ± 0.84	0.35 ± 0.12	3.71 ± 0.48	90.08 ± 1.22	1.74 ± 0.39

*SD* standard deviation, *TEC* transentorhinal cortex, *V*_*neu*_ volume fraction occupied by neurons, *V*_*g*_ volume fraction occupied by glia, *V*_*bv*_ volume fraction occupied by blood vessels, *V*_*n*_ volume fraction occupied by neuropil

The data for individual cases are shown in Additional file 1: Table S1

### Volume fraction of cortical elements

To estimate the possible degree of neuronal loss in the TEC in AD patients, the V_v_ was estimated for neurons, glia, blood vessels and neuropil. In control subjects, the volumes occupied by neuronal somata, glia somata, blood vessels and neuropil were 7.17%, 0.50%, 3.28% and 89.05%, respectively. In AD patients, these volumes were 5.86%, 0.35%, 3.71% and 90.08%, respectively. There was no significant difference between control and AD cases (MW, *p* > 0.05; Table 2; Additional file 1: Figure S1; Additional file 1: Table S1).

### Synaptic density

To compare synaptic features between control subjects and AD patients, synapses were examined in the neuropil from layer II of TEC (i.e., avoiding the cell bodies of neurons as well as glial and blood vessels; Additional file 1: Figure S2) [22]. In order to assure that FIB/SEM sampling was obtained from the same cortical layer, all the samples (from both control and AD subjects) were taken below the first row of neurons beneath layer I at a distance of approximately 267.03−506.10 μm from the surface. We refer to this as layer II regardless of whether or not layer III was distinguished.

**Fig. 5:**
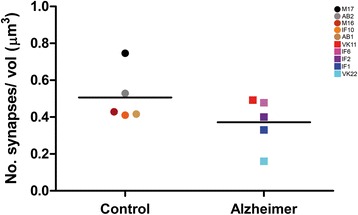
Graph showing the overall mean synaptic density in control and AD patients. Control cases are represented by circles and AD cases are represented by squares. Each color corresponds to each case analyzed in the study, as denoted in the upper right corner. No significant differences were found between groups (*p*-value > 0.05)

A total of 6102 synapses were identified; of these, 4646 synapses were analyzed —after discarding incomplete ones and those excluded by the counting frame— of which 2656 synapses were from control subjects (total tissue volume analyzed 5295 μm^3^), and 1990 were from AD patients (total tissue volume analyzed 5266 μm^3^) (Table 3, Additional file 1: Table S2). The number of synapses per volume was calculated by dividing the total number of synapses by the volume of the counting frame. Although values were lower in AD patients, we did not find significant differences in synaptic density (MW, *p* = 0.22) between control subjects (range: 0.41−0.75 synapses/μm^3^) and AD patients (range: 0.16−0.49 synapses/μm^3^; Fig. 5; Table 3, Additional file 1: Table S2).

**Table 3:** Accumulated data obtained from the ultrastructural analysis of neuropil from layer II of the TEC. All volume data are corrected for shrinkage factor

Group	No. AS synapses	No. SS synapses	No. all synapses	% AS Synapses (mean ± SD)	% SS Synapses (mean ± SD)	CF volume (μm^3^)	No. AS synapses/μm^3^ (mean ± SD)	No. SS synapses/μm^3^ (mean ± SD)	No. synapses/μm^3^ (mean ± SD)	Distance to nearest neighbor (nm; mean ± SD)
Control	2545	111	2656	95.64 ± 1.24	4.36 ± 1.24	5295	0.49 ± 0.14	0.02 ± 0.01	0.51 ± 0.14	791.54 ± 74.47
Alzheimer	1887	103	1990	94.47 ± 0.72	5.53 ± 0.72	5266	0.35 ± 0.14	0.02 ± 0.01	0.37 ± 0.15	881.50 ± 129.37

*All* includes AS+SS synapses, *AS* asymmetric synapses, *No.* number, *SD* standard deviation, *SS* symmetric synapses

The data for individual cases are shown in Additional file 1: Table S2

For practically all synaptic junctions found in control and AD patients, 3D segmentation allowed unambiguous classification into AS (prominent PSD) and SS (thin PSD) [29, 53, 54]. The proportion of each type of synapse was 95.64% (AS) and 4.36% (SS) in control subjects, and 94.47% and 5.53%, respectively, in AD patients (Table 3, Additional file 1: Table S2). Hence, no significant differences in proportions between control subjects and AD patients (MW, *p* = 0.15) were observed. Thus, the proportion of AS (excitatory) and SS (inhibitory) remained unchanged in layer II from TEC originating from AD patients.

### Synaptic morphology: Synaptic apposition surface (SAS)

Synaptic morphological analysis was performed by extracting the SAS from each synapse [50]. SAS features such as the area, the perimeter and the curvature showed no significant differences between groups, and this was the case for both AS and SS (MW, *p* > 0.05; Table 4, Additional file 1: Table S3). Frequency distribution analysis did not reveal significant differences either (KS, *p* > 0.05; Additional file 1: Figure S3). Values of SAS fitted to a log-normal distribution in both cases (Additional file 1: Figure S3). In summary, AD does not seem to affect the shape and size or the synaptic junctions.

**Table 4:** Area (nm^2^), perimeter (nm) and curvature (ratio) of the SAS. All data are corrected for shrinkage factor

Group	Type of synapse	Area of SAS (nm^2^; mean ± sem)	Perimeter of SAS (nm; mean ± sem)	Curvature of SAS (mean ± sem)
Control	AS	117,800 ± 1957	1653 ± 18.94	0.049 ± 0.001
	SS	76,190 ± 4082	1423 ± 49.14	0.050 ± 0.004
Alzheimer	AS	123,200 ± 2327	1697 ± 22.96	0.049 ± 0.001
	SS	67,750 ± 3611	1284 ± 41.00	0.049 ± 0.003

*AS* asymmetric synapses, *sem* standard error of the mean, *SAS* synaptic apposition surface, *SS* symmetric synapses

The data for individual cases are shown in Additional file 1: Table S3

### Synaptic distribution: 3D spatial analysis

To analyze the spatial distribution of synapses, we compared the actual position of the centroids of synaptic junctions with the CSR model. A random distribution follows the basic reference model of a CSR point process or homogeneous spatial Poisson point process. We calculated the G, F and K functions for the 30 samples from controls (*n* = 15) and AD patients (*n* = 15), comparing each one with 100 simulations of the CSR model. Results from these comparisons indicated a clear fit of the samples to a CSR model, since G, F and K functions closely resembled the theoretical curve that represents these functions of a homogeneous Poisson process, both in control subjects and in AD patients (Additional file 1: Figure S4). Note that in the G function there is a dead space (indicated with an arrow) due to the fact that synapses cannot be too close to each other since they cannot overlap in space.

In addition to the location of synapses in each sample, we measured the distance of each synapse to its nearest synapse. The mean distance to its nearest neighbor measured between centroids of synaptic junctions was 791.54 nm in control subjects and 881.50 nm in AD patients. No significant differences between groups (MW, *p* = 0.42; Table 3, Additional file 1: Table S2) were found.

Therefore, our analysis indicates that, the spatial organization of synapses in the neuropil of layer II of the TEC corresponds to a random distribution, regardless of the type of sample (control subjects or AD patients).

## Discussion

There were two main findings in the present study. First, at the light microscope level, we found that cortical thickness of the TEC displayed a severe reduction in AD patients, whereas no differences were observed in the volume occupied by neuronal and glial cell bodies, blood vessels and neuropil. Second, at the ultrastructural level, the analysis of the density, morphological features and spatial distribution of synapses in 3D electron microscope samples of layer II TEC neuropil from control and AD human brain samples showed no significant differences between groups.

Our data were derived from only five control cases and five AD patients. Therefore, the data obtained in the present study cannot be extrapolated to the whole population of patients with AD. However, although we examined relatively few cases, FIB/SEM samples do allow an unprecedented number of large stacks of serial ultrastructural images to be obtained from the neuropil, which provides 3D reconstructions of synapses to accurately determine their density, types, morphological features and spatial distribution in the human brain. Thus, the present results should be considered as robust data that should be verified in more cases and brain areas.

### Volume occupied by cortical elements: Loss of cells

No significant differences were found regarding the volume occupied by different cortical elements between AD and control cases. In particular, the volume fraction occupied by neurons was 7.17% in control samples and 5.86% in AD. However, considering the severe reduction of TEC thickness, the total number of neurons in AD patients would be expected to be lower than in control cases. That is, since the cortical thickness in AD patients is 35% thinner than in controls, and, in addition, we did not observe significant changes in the neuronal size in AD samples, the total number of neurons must be dramatically reduced. These results are in line with previous studies in the EC of AD patients reporting a loss of neurons [28, 86]. Similarly, a reduction of cortical thickness in AD has been previously reported in frontal cortex, in areas 21 and 22 of Brodmann, as well as in the molecular layer of DG [59, 60, 63, 69]. The reduction of TEC thickness observed in the present study was apparently unrelated to the abundance of NFTs and Aβ plaques, since patient IF1 showed a TEC thickness close to control values, but abundant NFTs and Aβ plaques in TEC (Figs. 2, 3), similar to other AD patients that showed a clear reduction in the TEC thickness. Further studies with additional AD patients would be necessary to assert the correlation between the TEC thickness and the presence of NFTs and Aβ plaques.

### Synaptic changes related to AD

Analysis of neuropil in the stacks of images of layer II of the TEC revealed that AD patients did not show a significantly lower synaptic density. However, since we found that TEC thickness in AD patients was 35% thinner than in control subjects, it follows that a decrease in the total number of synapses in AD occurs in this region. Previous studies performed in both AD patients and in animal models using light or electron microscopy to identify synapses —light microscopy immunocytochemical labeling of synaptic markers (mostly used in studies of human brains) or identification of synaptic junctions at the electron microscope level (see below)— have reported a loss of synapses per volume in the molecular layer of DG, CA1, temporal and cingulate gyrus, as well as in others regions of neocortex [2, 61, 62, 64–67]. However, other studies found an increase in synaptic density in neocortex and hippocampus from APP/PS1 mice [38], or no alterations in EC from AD patients [68]. Discrepancies might be attributable to specific characteristics of the animal models, or features of the analyzed regions, such as those that may be due to sampling differences. For example, near Aβ plaques there is a reduction in the number of synapses [25] and the density of plaques depends on several factors such as the animal model used and the age of the animals. Moreover, it is common to estimate synaptic density indirectly by counting —at the light microscopic level— immunoreactive puncta using synaptic markers [34, 44]. Quantification of synaptic density in single ultrathin sections using transmission electron microscopy to infer 3D characteristics of synaptic junctions observed in two dimensions [64] could be inaccurate for synaptic density estimations depending on the stereological method and other technical constraints (for further discussion, see [22, 45]). FIB/SEM technology has been proved to be an excellent tool to study the ultrastructure and alterations of synaptic organization of the human brain [8]. Using this technique, we were able to fully reconstruct synaptic junctions in a 3D volume of tissue, thus making possible the identification and classification of all synaptic junctions as AS or SS — thereby solving the technical limitations of other methodologies and obtaining more accurate data about the density of synapses [45]. Thus, the present results indicate that there is not a reduction in the number of synapses per volume of neuropil but —given that there is a decrease in thickness of TEC— it is obvious that there is a decrease in the *absolute* number of synapses in AD patients.

Nevertheless, we did not find differences in the proportion of AS and SS, suggesting that, in the neuropil, there is not an imbalance between excitatory and inhibitory circuits in layer II of the TEC. Since the reduction in the absolute number of synapses affected AS and SS equally, and in the cortex the majority of synapses are AS, the major decrease might be due to the loss of AS. It is well known that AS are mostly formed with dendritic spines of pyramidal cells [21]. This suggests that it is likely that dendritic spine disconnection or dendritic spine loss in the TEC of AD patients occurs. Our results are in agreement with the previously reported alterations of dendritic spines in AD patients [40, 47, 55]. However, since our data are derived from the study of the neuropil, we cannot rule out alterations in the axo-somatic or axo-axonic synapses (i.e., changes in the number, size and shape).

Synaptic changes observed in AD have been proposed to occur during early phases of the disease in subcortical regions and TEC, as these regions represent the areas that are first altered, particularly affecting the monoaminergic system [11, 12, 72]. However, since most AD patients examined in the present study correspond to advanced stages of the diseases, we do not know when the synaptic loss occurred. Aβ peptides and tau proteins play normal roles at the synapse, but under pathological conditions, they may produce toxic effects at both pre- and post-synaptic elements, leading to synaptic loss and causing dysfunction in neurotransmitter release [25, 32, 57, 73, 89]. Association of Aβ oligomers with synaptic structures in AD has been related to alterations in both synapses and dendritic spines [31, 39, 87]. Further analysis of the identity of post-synaptic elements (i.e., dendritic spines versus dendritic shafts) in layer II of the TEC is necessary in order to elucidate whether there are changes in the synaptic targets due to AD.

### Synaptic changes and interindividual variability

Despite the results regarding decrease in the number of synapses, it is important to note that there was remarkable interindividual variability. Technical effects were ruled out given that the postmortem delays were all similar and the procedures used were the same. We found that, in particular, two AD patients (IF6, VK11) showed synaptic density values similar to control subjects. According to the neuropathological criteria, these two patients presented NFTs only in the hippocampus and a low density of plaques (Braak/CERAD stage: III/A). By contrast, the AD patient who had the lowest synaptic density (VK22) presented NFTs widely distributed throughout the neocortex and a high density of plaques (Braak/CERAD stage: V/C). Thus, variability in synaptic density may be related to differences in the disease progression, that is, the more pathological signs, the less synaptic density. However, we also found differences in the synaptic density in AD cases with similar stages (Braak/CERAD stage: V/C), such as IF2 and VK22, whose synaptic densities were 0.40 synapses/μm^3^ and 0.16, respectively. In this regard, cognitive reserve has been proposed to account for the disjunction between the degree of brain pathology and its clinical manifestations [74]. This concept relies on the idea that individual differences in task processing may allow some individuals to cope better than others with brain changes, in general, including AD-related changes [75, 76]. Interestingly, case IF1 (Braak/CERAD stage: IV/B) apparently did not display cognitive impairment and, although its synaptic density was relatively low (0.33 synapses/μm^3^), the TEC had a greater thickness (2.3 mm) than cases with cognitive impairment. As pointed out by Ferrer [26], it should be kept in mind that AD—at least limited to the EC and TEC (stages I–II)— affects about 80% of individuals over 65 years, but dementia only occurs in a small percentage of individuals at this age (the prevalence of dementia in AD increases to 25% in 80-year-old individuals). Thus, it is possible that this particular case (IF1) may represent a pre-dementia stage of AD (prodromal AD) [26].

Nevertheless, there is an ongoing debate about the relationship of hyperphosphorylated-tau protein and the cognitive deficits in AD [16], due to the fact that the basic mechanism or mechanisms of cognitive deterioration are still not well understood.

In a previous study assessing the possible alterations to dendritic spines in pyramidal cells from AD patients [47], a remarkable loss of dendritic spines from pyramidal cells depending on the state of neurofibrillary pathology was found: in the so-called putative ‘pre-tangle’ stage, the dendritic trees of pyramidal neurons were unchanged. In the presence of well-developed NFTs, however, dendritic spine loss was obvious. In cases with an intermediate state of neurofibrillary pathology, the loss of dendritic spines was more variable. Since pyramidal neurons represent the principal building blocks of the cerebral cortex and dendritic spines are the main post-synaptic elements of cortical excitatory synapses and are fundamental structures in memory, learning and cognition [19], these alterations constitute what we think is an important event in the pathogenesis of AD. Therefore, the presence of hyperphosphorylated-tau protein in neurons does not necessarily mean that they suffer severe and irreversible effects as thought previously, but rather the characteristic cognitive impairment in AD is likely to depend on the relative number of neurons that have well-developed NFTs.

Finally, interindividual variability also emerges in the control group (synaptic density ranged from 0.41 to 0.75 synapses/μm^3^; see Additional file 1: Table S2). Although there are no studies in this brain region, previous studies on synaptic density in the temporal human cortex have shown variability depending on the gender (from 0.72 to 1.06 synapses/μm^3^) [1]. Furthermore, it has been found that some cortical areas of the macaque monkey display synaptic differences linked to aging [52]. Thus, although the number of cases analyzed in the present study is relatively low, the interindividual variability found in the present study might also be explained by gender or age differences.

### Analysis of the synaptic morphology

Synapses of layer II from TEC in AD patients showed similar morphological features to control cases. In particular, our results did not find any differences in the SAS area, perimeter and curvature. These results are also in line with some previous studies showing no changes in synaptic apposition length in AD patients [62, 68]. However, other studies performed in human tissue samples or animal models have shown an increase in synaptic apposition length, and these changes have been suggested to occur as a compensatory mechanism in response to the decrease in the number of synapses [2, 59, 60, 63, 69]. In this regard, it is important to point out that in the present study, we have measured the SAS instead of the synaptic apposition length because of its advantages over other methods. First, it is extracted automatically from the previously segmented synaptic junction with no user intervention, avoiding any manual tracing and possible associated user bias [2, 50]. Second, the SAS, despite being a surface, is also a 3D object that adapts to, and reproduces the shape and curvature of the PSD. Third, quantitative information on the surface area, perimeter and curvature can also be extracted from the SAS, so size and shape can easily be correlated. Thus, the present results obtained from a large number of synaptic junctions segmented in 3D (*n* = 4646 synapses) provide support for there being no change in the SAS parameters evaluated here.

It is well established that synapses are dynamic structures than can undergo modifications due to variations in the activity patterns, and they are continuously remodeled and replaced [54]. Thus, our ultrastructural analysis indicates that this synaptic dynamism may not be altered in AD. Furthermore, the shape and size of the synaptic junctions are strongly correlated with release probability, synaptic strength, efficacy and plasticity [7, 27, 33, 51, 77, 83, 84]. However, we cannot rule out changes in the composition of neurotransmitter receptors, ion-channels, structural and signaling proteins, etc. For example, comparative human post-synaptic density (PSD) proteome analysis between control and AD patients has shown changes in several proteins involved in various cellular functions [17, 88]. Moreover, some loss of dendritic spines may occur in layer II of the TEC in AD or some dendritic spines may turn into non-synaptic dendritic spines [41, 47]. Thus, although no changes in the number of synapses were found in the present study, it is possible that a reorganization of synaptic targets (i.e., changes in the proportion of axo-spinous versus axo-dendritic shafts synapses) may occur in AD.

### Synaptic distribution: 3D spatial analysis

The spatial distribution of synapses can be distributed according to 3 patterns: random pattern (where each synapse is equally likely to occur at any position); regular pattern (where each synapse is located as far as possible from its neighbors); and clustered pattern (where synapses tend to concentrate in groups). Moreover, when modeling the spatial distribution of synapses, we should take into account the fact that synaptic junctions cannot overlap because they are independent objects, and thus the minimum intersynaptic distances must be limited by the size of the synaptic junctions themselves [46].

Spatial distribution analysis showed that in both control and AD groups, synapses in the neuropil follow a random spatial distribution. Previous studies on the spatial distribution of synapses in plaque-free regions of neuropil from the frontal cortex in AD patients, in the molecular layer of DG from APP/PS1 mice, and in control somatosensory cortex of rat have also found a random pattern distribution [2, 8, 46]. Thus, it seems that random distribution of synapses is a widespread rule of the cerebral cortex that does not appear to be affected in AD.

In addition to the analysis of the spatial distribution of synapses, we examined the distance of each synapse to its nearest synapse and we did not find significant differences between control subjects and AD patients. The lowest mean distance to the nearest synapse (678.91 nm) corresponds to the subject with the highest synaptic density, while the highest mean distance to the nearest synapse (1102.29 nm) belongs to the AD patient showing the lowest synaptic density. Thus, despite synaptic variability found between subjects, it seems that lower synaptic densities are related to higher distances. Since we found a significant reduction in TEC cortical thickness and not significant differences in the synaptic density, we would expect a reduction in the distance to the nearest synapse in AD patients. However, this distance was slightly higher (but not significant) supporting the notion of a reduction in the overall number of synapses in layer II of TEC in these patients.

## Conclusions

In summary, the present results provide support for neuronal and synaptic loss also occurring in the TEC, in line with previous studies. However, this decrease is not due to a reduction in the synaptic density, but it is inferred from the reduction in TEC cortical thickness. Since it is likely that there is shrinkage of the dendritic arborization of neurons concomitant with the reduced cortical thickness, there must be a compensatory mechanism (for example, generation of new dendritic branches) in the surviving or healthy neurons in order to explain the lack of changes in synaptic density. Finally, whether or not synaptic loss precedes neuronal loss by synaptic disconnection is not known, but our previous studies on the morphological alterations in dendritic spines of pyramidal cells in AD patients [47] seem to provide strong support for synaptic loss preceding neuronal loss.

## Additional file

Additional file 1: **Figure S1.** Estimation of the volume occupied by cells, blood vessels and neuropil using the method of Cavalieri. **Figure S2.** Serial images obtained by FIB/SEM from the neuropil of layer II of TEC from an Alzheimer’s disease patient. **Figure S3.** Frequency plots of synaptic apposition surface (SAS) distribution. **Figure S4.** Analysis of the 3D synaptic spatial distribution. **Table S1.** Light microscopy data on volume fraction occupied by cortical elements in layer II of the TEC. **Table S2.** Data from the ultrastructural analysis of neuropil from layer II of the TEC. **Table S3.** Data regarding area (nm2), perimeter (nm) and curvature (ratio) of the SAS by individual cases. (PDF 1216 kb)

## Abbreviations

3D: Three-dimensional
AD: Alzheimer's disease
APP/PS1: Amyloid precursor protein/presenilin-1 mouse model of Alzheimer’s disease
AS: Asymmetric synapses
Aβ: Amyloid-β
CA1: *Cornu ammonis* 1
CSR: Complete Spatial Randomness
DG: Dentate gyrus
EC: Entorhinal cortex
FIB/SEM: Focused ion beam/scanning electron microscopy
KS: Kolmogorov-Smirnov
MTL: Medial temporal lobe
MW: Mann-Whitney
NFTs: Neurofibrillary tangles
PB: Phosphate buffer
PRC: Perirhinal cortex
PSD: Post-synaptic density
SAS: Synaptic apposition surface
SD: Standard deviation
sem: Standard error of the mean
SS: Symmetric synapses
TEC: Transentorhinal cortex
V_v_: Volume fraction
