# Supplementary material for: Three-dimensional analysis of synapses in the transentorhinal cortex of Alzheimer’s disease patients
Source: Acta Neuropathol Commun. 2018 Mar 2;6:20. doi: 10.1186/s40478-018-0520-6 (PMC5834884; doi:10.1186/s40478-018-0520-6)
Supplement: Supplementary file 1 — Figure S1. Estimation of the volume occupied by cells, blood vessels and neuropil using the method of Cavalieri. Figure S2. Serial images obtained by FIB/SEM from the neuropil of layer II of TEC from an Alzheimer’s disease patient. Figure S3. Frequency plots of synaptic apposition surface (SAS) distribution. Figure S4. Analysis of the 3D synaptic spatial distribution. Table S1. Light microscopy data on volume fraction occupied by cortical elements in layer II of the TEC. Table S2. Data from the ultrastructural analysis of neuropil from layer II of the TEC. Table S3. Data regarding area (nm2), perimeter (nm) and curvature (ratio) of the SAS by individual cases. (PDF 1216 kb) [file 40478_2018_520_MOESM1_ESM.pdf]

Three-Dimensional Analysis of Synapses in the Transentorhinal Cortex of Alzheimer´s Disease patients

Domínguez-Álvaro M[1], Montero-Crespo M[1,2], Blázquez-Llorca L[1,5], Insausti R[4], DeFelipe J[1,2,3], Alonso-Nanclares L*[1,2,3]

[1]Laboratorio Cajal de Circuitos Corticales, Centro de Tecnología Biomédica, Universidad Politécnica de Madrid. Pozuelo de Alarcón, 28223 Madrid, Spain; [2]Instituto Cajal, Consejo Superior de Investigaciones Científicas (CSIC) Avda. Doctor Arce, 37 Madrid, 28002, Spain; [3]Centro de Investigación Biomédica en Red sobre Enfermedades Neurodegenerativas (CIBERNED), ISCIII, Madrid, Spain; [4]Laboratorio de Neuroanatomía Humana, Facultad de Medicina, Universidad de Castilla-La Mancha, Almansa 14, Albacete 02006, Spain; [5]Depto. Psicobiología, Facultad de Psicología, Universidad Nacional de Educación a Distancia (UNED) c/Juan del Rosal, 10 Madrid, 28040, Spain;

*Correspondence: L. Alonso-Nanclares (aidil@cajal.csic.es)

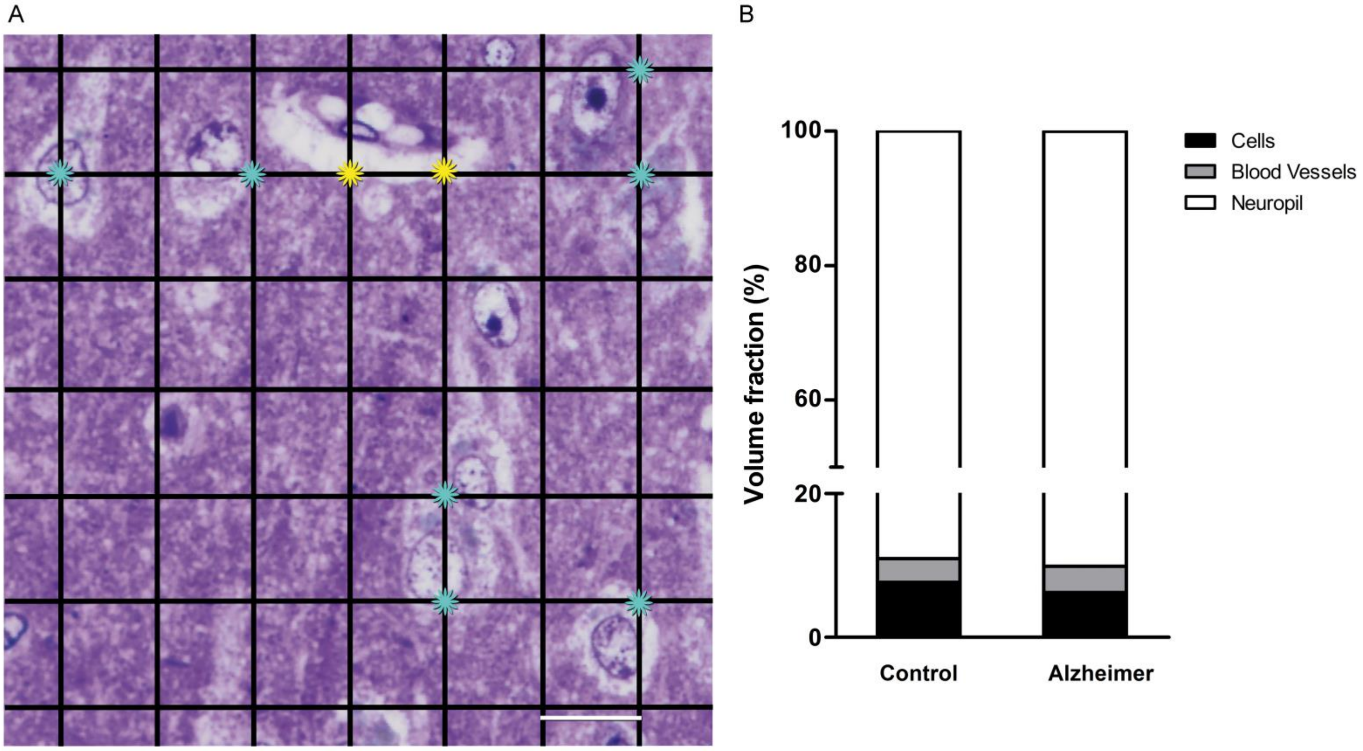

Supplementary Fig. 1 Estimation of the volume occupied by cells, blood vessels and neuropil using the method of Cavalieri. Photomicrograph of a semithin section (2µm) stained with toluidine blue, showing layer II of the TEC from a subject with no apparent neurological alterations **(A)**. A grid of small intersections overlying the tissue is displayed. Each grid point covers an associated area of 20x20=400µm^2. Blue asterisks indicate the intersections in the grid that lie within neuronal bodies. Yellow asterisks indicate the intersections in the grid that lie within blood vessels **(A)**. Graph showing the volume fraction occupied by all cells (neuron + glia), blood vessels and neuropil in layer II of the TEC from control subjects and AD patients, indicating no differences between the two groups **(B)**. Scale bar (in A): 20µm.

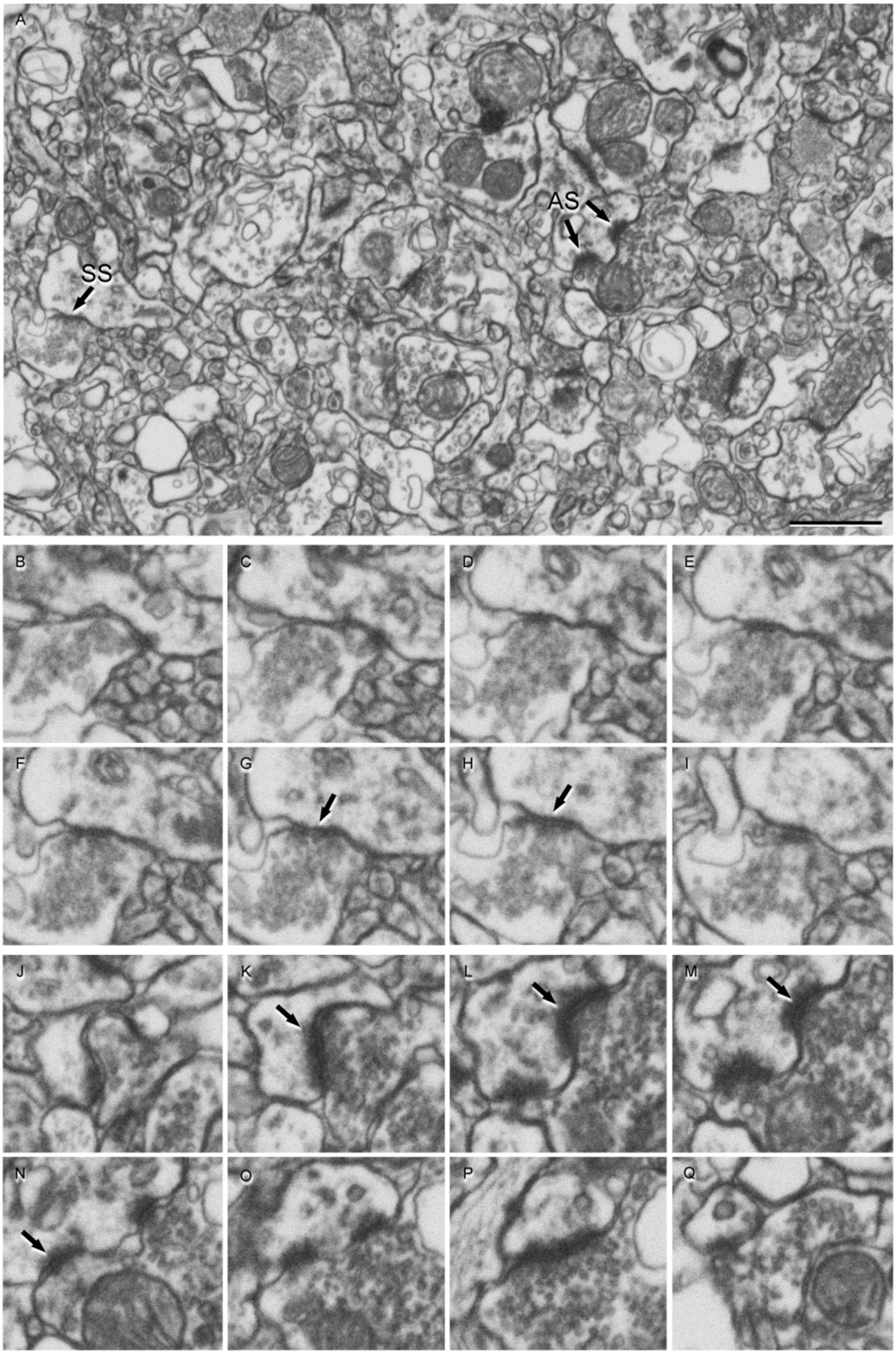

Supplementary Fig. 2 Serial images obtained by FIB/SEM from the neuropil of layer II of TEC from an Alzheimer's disease patient. Low magnification photograph showing numerous synapses **(A)**, two of which have been selected as examples (black arrows) of asymmetric (AS) and symmetric synapses (SS). Synapse classification was based on the examination of the full sequence of serial images; an SS can be visualized in B–I, and an AS in J–Q. Scale bar (in A): 1000nm in A; 500nm in B–Q.

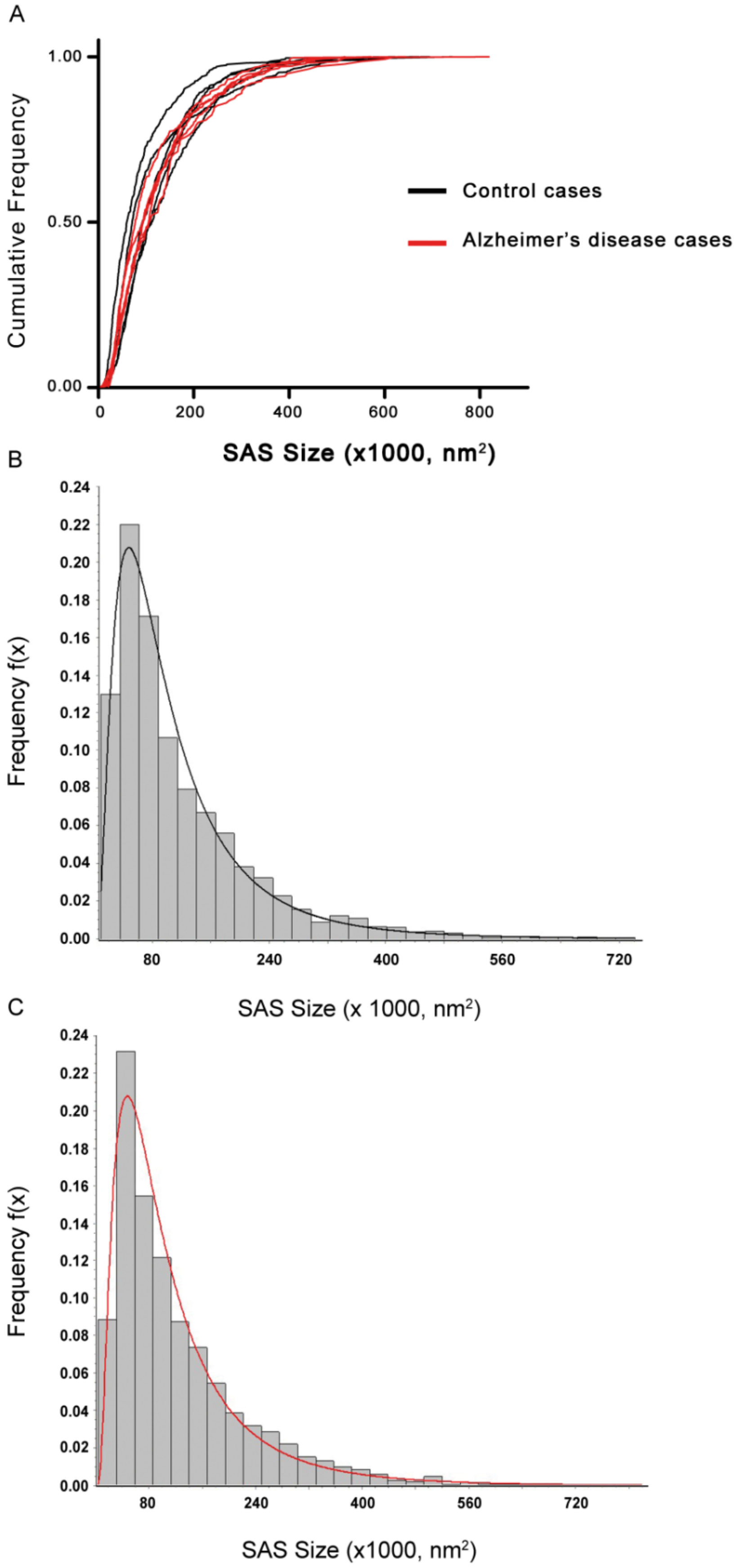

Supplementary Fig. 3 Frequency plots of synaptic apposition surface (SAS) distribution. Cumulative frequency plots showing the distribution of the SAS per sample in control (black lines) and Alzheimer's disease cases (red lines; **A**). Frequency distribution histograms of the SAS area in control group **(B)** and Alzheimer's disease group **(C)**. In both groups, values of SAS area fit to a log-normal distribution.

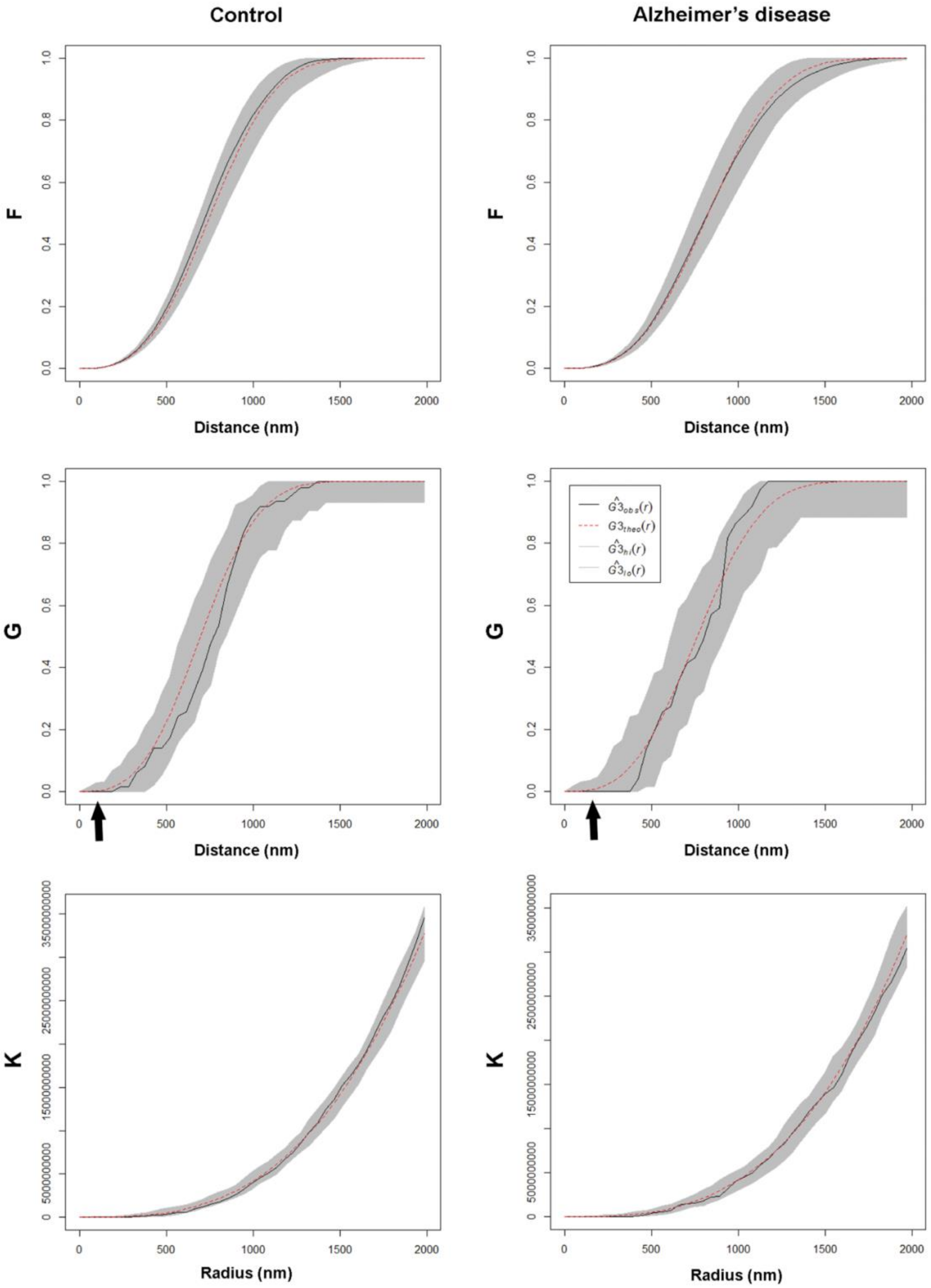

Supplementary Fig. 4 Analysis of the 3D synaptic spatial distribution. Red dashed traces correspond to a theoretical homogeneous Poisson process for each function. The black continuous traces correspond to the experimentally observed function. The shaded areas represent the envelopes of values calculated from a set of 99 simulations. All plots show a distribution which fit into a Poisson function. In the G function, the arrow shows a dead space due to the fact that synapses cannot be too close to each other since they cannot overlap in space. F: F function; G: G function; K: K function.

Supplementary Table 1. Light microscopy data on volume fraction occupied by cortical elements in layer II of the TEC. All volume data are corrected for shrinkage. TEC: transentorhinal cortex; V_{neu}: volume fraction occupied by neurons; V_g: volume fraction occupied by glia; V_{bv}: volume fraction occupied by blood vessels; V_n: volume fraction occupied by neuropil.

**Case**	**V_{neu} (%; mean± SD)**	**V_g (%; mean± SD)**	**V_{bv} (%; mean± SD)**	**V_n (%; mean± SD)**	**TEC thickness (mm; mean± SD)**
**AB1**	6.13±0.31	0.26±0.03	2.75±0.04	90.86±0.06	2.74±0.08
**AB2**	8.02±1.25	0.52±0.02	3.08±1.26	88.38±1.68	2.56±0.09
**IF10**	6.28±0.05	0.61±0.01	3.77±0.06	89.34±0.07	2.06±0.05
**M16**	8.29±1.09	0.51±0.02	3.61±1.18	87.59±1.64	2.96±0.06
**M17**	7.13±1.08	0.59±0.01	3.19±0.03	89.09±0.09	2.96±0.06
**IF1**	5.01±0.05	0.27±0.01	2.96±0.01	91.76±0.05	2.26±0.12
**IF2**	5.46±0.05	0.54±0.01	3.60±0.02	90.40±0.08	1.20±0.03
**IF6**	7.07±0.07	0.26±0.00	3.89±0.04	88.78±1.10	1.89±0.10
**VK11**	5.39±0.06	0.32±0.01	3.81±0.04	90.48±0.06	1.79±0.06
**VK22**	6.37±0.08	0.37±0.01	4.26±0.03	89.00±1.01	1.57±0.09

Supplementary Table 2. Data from the ultrastructural analysis of neuropil from layer II of the TEC. All volume data are corrected for shrinkage factor. All: includes AS+SS synapses; AS: asymmetric synapses; No.: number; SD: standard deviation; SS: symmetric synapses.

Case	No. AS synapses	No. SS synapses	No. all synapses	% AS Synapses (mean± SD)	% SS Synapses (mean± SD)	CF volume (μm^3)	No. AS synapses/μm^3 (mean± SD)	No. SS synapses/μm^3 (mean± SD)	No. synapses/μm^3 (mean± SD)	Distance to nearest neighbor (nm; mean± SD)
**AB1**	409	25	434	94.12±1.35	5.88±1.35	1045.20	0.39±0.03	0.02±0.01	0.41±0.03	812.32 ±33.31
**AB2**	432	13	445	97.24±1.55	2.76±1.55	819.64	0.51±0.05	0.02±0.01	0.53±0.05	757.95±17.24
**IF10**	418	22	440	94.99±0.58	5.01±0.58	1057.05	0.40±0.01	0.02±0.00	0.42±0.01	860.07 ±19.27
**M16**	575	26	601	95.31±1.51	4.69±1.51	1389.27	0.41±0.08	0.02±0.00	0.43±0.08	848.46±88.77
**M17**	711	25	736	96.52±0.62	3.48±0.62	983.53	0.72±0.09	0.03±0.00	0.75±0.09	678.91±23.33
**IF1**	349	23	372	93.77±2.20	6.23±2.20	1122.73	0.31±0.01	0.02±0.01	0.33±0.02	891.30±41.18
**IF2**	439	21	460	95.29±1.36	4.71±1.36	1156.28	0.38±0.09	0.02±0.00	0.40±0.09	816.73±34.19
**IF6**	430	25	455	94.20±2.38	5.80±2.38	958.76	0.45±0.11	0.03±0.01	0.48±0.10	807.28 ±86.63
**VK11**	533	25	558	95.19±1.96	4.81±1.96	1127.98	0.47±0.14	0.02±0.01	0.49±0.14	789.90±111.26
**VK22**	136	9	145	93.88±1.18	6.12±1.18	899.87	0.15±0.02	0.01±0.00	0.16 0.02	1102.29±105.22

Supplementary Table 3. Data regarding area (nm^2), perimeter (nm) and curvature (ratio) of the SAS by individual cases. All data are corrected for shrinkage factor. AS: asymmetric synapses; sem: standard error of the mean; SAS: synaptic apposition surface; SS: symmetric synapses.

Group	Type of synapse	Area of SAS (nm^2; mean± sem)	Perimeter of SAS (nm; mean± sem)	Curvature of SAS (mean± sem)
**AB1**	AS	140718±5250	1786±47.12	0.047±0.002
	SS	73839±9793	1354±111.50	0.035±0.003
**AB2**	AS	85838±3842	1418±42.97	0.051±0.002
	SS	48329±7450	1346±164.20	0.101±0.016
**IF10**	AS	128162±4403	1668±35.08	0.043±0.002
	SS	83442±10768	1428±104.90	0.033±0.003
**M16**	AS	116514±3509	1623±32.09	0.044±0.002
	SS	84247±8770	1514±110.20	0.056±0.011
**M17**	AS	118951±4290	1740±44.93	0.055±0.001
	SS	78095±6095	1431±86.44	0.049±0.005
**IF1**	AS	122257±4738	1632±44.62	0.049±0.002
	SS	85542±8020	1497±96.34	0.042±0.004
**IF2**	AS	125035±4725	1668±40.90	0.051±0.002
	SS	50771±5929	1176±81.00	0.066±0.007
**IF6**	AS	116269±5171	1683±54.83	0.045±0.002
	SS	81554±8538	1384±83.20	0.039±0.004
**VK11**	AS	122618±4278	1774±48.42	0.051±0.001
	SS	53956±5388	1111±69.93	0.054±0.007
**VK22**	AS	143809±10586	1695±69.59	0.049±0.002
	SS	61927±8655	1193±102.9	0.041±0.006
